# Identification of Alternative Splicing-Related Genes CYB561 and FOLH1 in the Tumor-Immune Microenvironment for Endometrial Cancer Based on TCGA Data Analysis

**DOI:** 10.3389/fgene.2022.770569

**Published:** 2022-06-28

**Authors:** Dan Sun, Aiqian Zhang, Bingsi Gao, Lingxiao Zou, Huan Huang, Xingping Zhao, Dabao Xu

**Affiliations:** Department of Gynecology, Third Xiangya Hospital of Central South University, Changsha, China

**Keywords:** alternative splicing, tumor-immune microenvironment, endometrial cancer, CYB561, FOLH1

## Abstract

**Background:** Advanced and recurrent endometrial cancer EC remains controversial. Immunotherapy will play a landmark role in cancer treatment, and alternative splicing (AS) of messenger RNA (mRNA) may offer the potential of a broadened target space.

**Methods:** We downloaded the clinical information and mRNA expression profiles from The Cancer Genome Atlas (TCGA) database. Hub genes were extracted from 11 AS-related genes to analyze the correlation between clinical parameters and the tumor-immune microenvironment. We also analyzed the correlations between the copy numbers, gene expressions of hub genes, and immune cells. The correlation between the risk score and the six most important checkpoint genes was also investigated. The ESTIMATE algorithm was finally performed on each EC sample based on the high- and low-risk groups.

**Results:** The risk score was a reliable and stable independent risk predictor in the Uterine Corpus Endometrial Carcinoma (UCEC) cohort. CYB561|42921|AP and FOLH1|15817|ES were extracted. The expression of CYB561 and FOLH1 decreased gradually with the increased grade and International Federation of Gynecology and Obstetrics (FIGO) stage (*p* < 0.05). Gene copy number changes in CYB561 and FOLH1 led to the deletion number of myeloid DC cells and T cell CD8^+^. Low expression of both CYB561 and FOLH1 was associated with poor prognosis (*p* < 0.001). The checkpoint genes, CTLA-4 and PDCD1, exhibited a negative correlation with the risk score of AS in UCEC.

**Conclusion:** AS-related gene signatures were related to the immune-tumor microenvironment and prognosis. These outcomes were significant for studying EC’s immune-related mechanisms and exploring novel prognostic predictors and precise therapy methods.

## Introduction

Uterine corpus endometrial carcinoma (UCEC) is one of the three major malignancies of the female reproductive system (Bray et al., 2018). Although most patients are diagnosed at an early stage and have a good prognosis, there are still some patients who are at an advanced stage at first diagnosis or have recurrence and metastasis after treatment, with a 5-year survival rate of only 20–26% (Morice et al., 2016). Therefore, further studies on therapeutic monitoring and prognostic assessment of UCEC are crucial for both clinicians and patients. In 2013, tumor immunotherapy was regarded as an important scientific breakthrough and suggested that immunotherapy will play a landmark role in the field of cancer treatment (Couzin-Frankel, 2013). In recent years, immune checkpoint inhibitors have made breakthrough progress and have been written into the treatment guidelines for endometrial cancer (EC).

Alternative splicing (AS) is a universal mechanism to produce mRNA isomers using a limited set of genes, resulting in structurally and functionally different protein isoforms, modifying more than 95% of human genes (Gilbert, 1978; Buratti et al., 2006). Studies have shown that aberrant AS is closely associated with the occurrence, development, metastasis, and drug resistance of various cancers (de Necochea-Campion et al., 2016; Climente-González et al., 2017; Wang and Lee, 2018; Bonnal et al., 2020). AS has also become a hot topic in tumor immunotherapy and attracted the attention of researchers. Several forms of mRNA processing are dysregulated in cancer and offer promise concerning immunotherapy target expansion (Venables, 2004; Baralle and Giudice, 2017; Kahles et al., 2018). Although significant progress has been achieved in expanding the immunotherapy target space using tumor-specific mRNA processing events, much work is still needed (Frankiw et al., 2019).

What’s more, splice factors might play a vital carcinogenic role in EC (Dou et al., 2020a; Li et al., 2020; Popli et al., 2020). By analyzing the whole genome of AS events in EC, studies have found several candidate splicing factors that may become therapeutic targets and predict patients’ prognosis by constructing gene signatures (Wang C. et al., 2019; Wang Q. et al., 2019), which further demonstrates the importance of AS events in EC. A study found that with the increase of the ESTIMATE score and the infiltration of immune cells in UCEC patients, the prognosis would be better (Liu et al., 2021); however, further discussion was lacking.

Given the importance of immunotherapy in UCEC, characterization of immune infiltrating features is essential for further understanding the oncogenesis of UCEC and the development of a novel prognostic signature and therapeutic response classifier. In the present study, whole-genome analysis and prognostic model construction were firstly used to determine prognosis-related genes involved in the AS prognostic model. The characteristics of two AS-related genes in the tumor-immune microenvironment were then analyzed. Finally, the correlation between the six most important immune checkpoint genes and the risk score was also investigated. Our research provides a more comprehensive insight into precise immunotherapy for UCEC.

## Methods

### Data Acquisition and Curation Processing

We downloaded the mRNA expression profiles and corresponding clinical data of the UCEC cohort from the TCGA database (June 2021, https://portal.gdc.cancer.gov/). The AS event data for UCEC were obtained from the https://bioinformatics.mdanderson.org/TCGASpliceSeq/(Ryan et al., 2016). Since these data are publically available, there was no requirement for approval by an ethics committee. We fully assessed the availability of clinical information. In our research, a few patients were excluded because they met the following criteria: 1) Epithelial neoplasm disease type, nos in TCGA; and 2) incomplete clinical data (e.g., age, grade, FIGO stage, and survival data). The percent spliced in (PSI) value can be used to quantify each AS event, which is the ratio of normalized reads indicating the presence of a transcript element versus the total normalized reads for that event, with a rating from 0 to 1. PSI = splice in/splice in+splice out. We screened the AS data for PSI value >0.75, representing the association between gene expression and AS events. We then merged the gene expression and clinical profiles using Perl (v5.30.0, https://www.perl.org/), establishing genomics and clinical databases for further research. A total of 524 patients with complete AS events and clinical data were included in our analysis. The clinical features of the patients are summarized in Table 1.

**TABLE 1:** The key demographic, clinical, and pathological characteristics of the 524 patients with UCEC.

Variables	Count	Percentage (%)
Age (mean ± SD)	63.88 ± 11.20	
Follow-up (mean ± SD) (y)	3.05 ± 2.47	
Status		
Alive	436	83.21
Dead	88	16.79
Histological type		
Adenomas and adenocarcinomas	390	74.43
Cystic, mucinous, and serous neoplasms	134	25.57
FIGO stage		
I	330	62.98
II	47	8.97
III	119	22.71
IV	28	5.34
Grade		
G1	93	17.75
G2	118	22.52
G3	313	59.73
Race		
White	361	68.89
Black or African American	104	19.85
Asian	19	3.63
Other	11	2.10
Not reported	29	5.53

UCEC: uterine corpus endometrial carcinoma; FIGO, international federation of gynecology and obstetrics.

### Screening for Prognostic AS Events in UCEC

TCGA SpliceSeq is a database based on TCGA RNA sequencing (RNA-seq) data. Seven types of selective splicing events were analyzed, including Alternate Acceptor site (AA), Alternate Donor site (AD), Alternate Promoter (AP), Alternate Terminator (AT), Exon Skip (ES), Mutually Exclusive Exons (ME), and Retained Intron (RI). We analyzed the distributions of all encoded genes using the UpSetR package in each of the seven different types of AS events and survival-related AS events in UCEC.

### Construction of Prognostic Models and Survival Analysis

Different AS events in genes led to diversity in outcomes, and changes in gene expression affected survival time. To further understand the prognostic value of AS events in UCEC patients, univariate Cox regression analysis with R package “survival” was performed to determine the survival-related different expressed alternative splicing (DEAS) events, including overall survival (OS)-related DEAS events. Next, the least absolute shrinkage and selection operator (LASSO) regression was applied to identify the final elimination of potential predictors with non-zero coefficients using the R package “glmnet”, which can avoid model overfitting to obtain a better fitting model. Furthermore, based on the results of LASSO Cox regression, predictive models were constructed using multivariate Cox regression analysis. Based on the PSI values and multivariate Cox analysis, we calculated the risk scores of each patient and obtained the corresponding coefficients, respectively. The following formula obtained the risk score:

$$\text{Risk}\;\text{score}=\sum_{i=0}^{n}\text{PSI}\times \beta_{\text{i}}$$

where *β* is the regression coefficient of the AS events. A total of 524 EC patients were divided into high- and low-risk groups bound by the median of risk score, and Kaplan-Meier survival analysis was performed to determine whether they had completely different prognoses. Furthermore, receiver operating characteristic (ROC) curves of 1, 3, and 5 years were generated using the survival ROC package in R to show the discrimination of the predictive signatures (Heagerty et al., 2000).

### Establishment and Validation of a Predictive Nomogram

All clinical factors, including the risk score, age, FIGO stage, and grade, were incorporated to construct a nomogram to evaluate the probability of 1-, 3-, and 5-year OS of UCEC patients in the entire set. Validation of the nomogram was evaluated by calibration plot using the “rms” package. The calibration curve of the nomogram was plotted to assess the nomogram-predicted probabilities against the actual rates.

### Immune Score Estimate and Immune Cell Infiltrating Proportion Inference

Normalized RNA expression data were used to infer the Immune Score using the estimate package (Yoshihara et al., 2013) and quantify the infiltrating proportions of 22 types of immune cells using the “CIBERSORT” package (Newman et al., 2015). The infiltrating percentage of 22 types of immune cells was equal to 100%. Single sample gene set enrichment analysis (ssGSEA) was used to quantify and classify the immunity stage based on immune-related gene (IRGs) sets (He et al., 2018). Next, 47 immune checkpoint genes were analyzed, and 16 of them that differed from the tumor and normal samples were screened. The differences between the 16 hub immune checkpoints among the high- and low-risk groups were analyzed, and the correlations between the six most important immune checkpoint genes (CD274, PDCD1, PDCD1LG2, CTLA4, HAVCR2, and IDO1) and the risk score were determined.

### Extraction of AS-Related DEGs in UCEC Samples

Using R package “limma” with the threshold of |log2FC|>1 and *p* < 0.05, the 11 genes (Table 2) involved in the model construction were analyzed to observe whether their expression differed between the UCEC and normal samples.

**TABLE 2:** Eleven AS events associated with the OS of UCEC patients.

ID	Coefficient	HR	HR.95L	HR.95H	*p* value
MAST1|47878|AT	1.756261	5.790747	2.208989	15.180133	0.000355
CYB561|42921|AP	2.851763	17.318280	3.689684	81.286860	0.000301
MAGED1|89145|AP	2.280885	9.785341	0.965560	99.168280	0.053569
PCYT2|44230|ES	1.310632	3.708517	1.054692	13.039925	0.041056
SULT1A3|94136|AP	−1.203415	0.300167	0.070887	1.271038	0.102203
FOLH1|15817|ES	−5.303092	0.004976	0.000452	0.054736	0.000015
ZNF706|84749|ES	3.998052	54.491877	2.482313	1196.208995	0.011185
CCNL2|162|ES	1.824693	6.200888	0.853256	45.063858	0.071366
RPLP0|24731|ES	−7.978477	0.000343	0.000003	0.043102	0.001218
STK32C|13483|AP	2.122228	8.349723	0.979867	71.150383	0.052215
C4orf29|70557|AT	1.696728	5.456064	0.611076	48.715079	0.128758

### Integration of AS-Related DEGs With Clinical Characteristics and Prognosis

High- and low-expression groups of gub genes were obtained according to the gene expression. These were then used to analyze the difference in clinical indicators, including age, grade, and FIGO stage. Finally, the prognosis of the hub genes in the two groups was judged using the “survival” and “survminer” packages.

### Analysis of the Relationship Between Stromal/Immune Scores and AS-Related DEGs in the EC Immune Microenvironment

The ESTIMATE algorithm was applied to analyze the Stromal Score, Immune Score, ESTIMATE Score, and Tumor Purity based on the transcriptome profiles of UCEC to determine the effect ssGSEA grouping. We further compared the Stromal Score, Immune Score, ESTIMATE Score, and Tumor Purity in the high- and low-expression groups of hub genes using the Limma.R and ggpubr.R packages. The relationships between the copy number of hub genes and the quantity of six immune cells (B cell, myeloid DC cell, macrophage, neutrophil, T cell CD4^+^, and T cell CD8^+^) were evaluated using the Tumor Immune Estimation Resource (TIMER) database.

### Construction of a Potential SF-AS Regulatory Network

Splicing factors (SF) are protein factors involved in the splicing process of RNA precursors, which are closely related to the development and treatment of cancer (Dvinge et al., 2016; Obeng et al., 2019). A total of 404 SFs data downloaded from the SpliceAid2 database were used to analyze the correlation between the expression level of SFs and the PSI values of OS-associated AS events by R packages (BiocManager, limma). An absolute value of the correlation coefficient >0.6 and *p* < 0.001 were considered statistically significant. Finally, Cytoscape software (v3.7.2, https://cytoscape.org/) was used to visualize the potential SF-AS regulatory network.

### Statistical Analysis

All statistical analyses were performed using R version 4.1.0 (R packages: survival, survminer, UpSetR, glmnet, estimate, ggpubr, e1071, rms, preprocessCore, vioplot, ggExtra, GSVA, GSEABase, reshape2, pheatmap, corrplot, ggplot, ggplot2, and BiocManager). For all analyses, a two-tailed *p* < 0.05 was regarded as statistically significant if not noted.

## Results

### Overview of AS Events in TCGA UCEC Cohort

**FIGURE 1:**
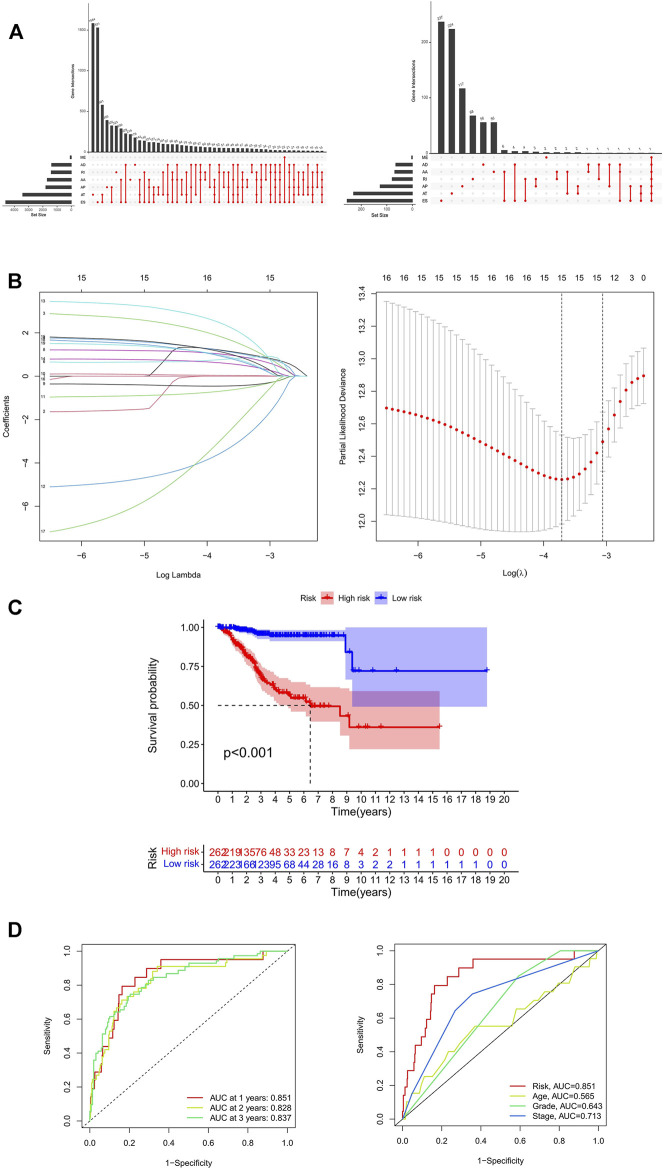
Identification and prognosis of AS markers in UCEC. **(A)** The upSet plot of intersections and aggregates among diverse types of AS (left) and survival-associated AS events (right) in UCEC. **(B)** LASSO coefficient profiles of survival-associated AS events and 10-time cross-validation for tuning parameter selection in the LASSO model. **(C)** Kaplan-Meier analysis for OS of UCEC patients. **(D)** ROC curve in the predicted groups (high- and low-risk groups) by the 11-AS events signature in the UCEC cohort. AA, alternate acceptor; AD, alternate donor; AP, alternate promoter; AT, alternate terminator; ES, exon skip; ME, mutually-exclusive exons; RI, retained intron; UCEC, Uterine Corpus Endometrial Carcinoma.

A total of 524 UCEC patients were identified, and the baseline characteristics of these patients are summarized in Table 1. The mRNA splicing data included in this study contains 28,281 AS events in 8,141 genes. Given the possibility of multiple splicing modes for a single gene, we created UpSet plots to analyze interactive sets of seven types of AS events quantitatively. As shown in Figure 1A, a single gene could have up to five different splicing modes, and most genes had more than one AS event. Exon skip (ES) was the most frequent splice type among the seven AS types (34.4%), followed by an alternate terminator (AT) (27.5%) and alternate promoter (AP) (15.7%).

### Prognostic Index Models Featured by AS Events for UCEC

To explore the prognostic utility of an AS signature in EC, AS events associated with OS were identified by fitting univariate Cox proportional hazard regression models after merging the clinical data in the training cohort using Perl. In total, 1,108 AS events were determined with *p* < 0.05, including 633 high-risk survival-associated AS events (hazard ratio HR > 1) and 475 low-risk survival-associated AS events (HR < 1). The AS events can be counted through the UpSet plot. An UpSet plot was generated to visualize the intersecting sets between different genes and AS events. The bar charts on the left showed the number of genes with some kind of AS events. The upper bar charts showed the number of intersecting genes, indicating the number of genes with a certain type or types of AS events (Figure 1A). Figure 1A indicates that one gene might have more than one survival-associated AS event. It is noteworthy that the three highest frequency survival-associated AS events were still ES, AT, and AP in the UCEC cohort.

After conducting univariate regression analysis, LASSO regression was performed to select the optimal survival-related AS events to construct the prediction models to avoid model overfitting based on OS. First, 15 AS events were screened out by LASSO regression, and then the AS events with the same contribution were optimized (Figure 1B). Finally, an 11-AS event signature was identified as a predictor of survival in EC through the Cox proportional hazards regression model (Table 2). Besides, the minimum adjusted estimate of cross-validation prediction error was 0.020, and the *p* value of bootstrap (*K* = 1000) was 4.82E-305.

Kaplan-Meier curves and log-rank tests were plotted to explore the relationship between risk score and survival status. The survival probability of low-risk patients was higher than that of high-risk patients; in other words, high-risk patients had a higher mortality rate, as illustrated in Figure 1C (*p* < 0.001). We then applied ROC analysis to compare the predictive power of these prognostic models. The larger the area under the curve, the higher the accuracy of the model to predict the prognosis of patients. Figure 1D showed a robust and significantly improved performance; the areas under the ROC curve (AUC) in 1, 2, and 3 years were all greater than 0.800. The result illustrated that the accuracy of using the model to predict the 1-, 2- and 3-year survival rate of patients was relatively high. Moreover, the AUC of the risk score model predicting the 1-year survival rate was larger than that of the age, grade, and FIGO stage (Figure 1D). It means that the accuracy of predicting the 1-year survival rate of patients by the model is better than that of using other clinical parameters (age, grade, stage) to predict the prognosis.

**FIGURE 2:**
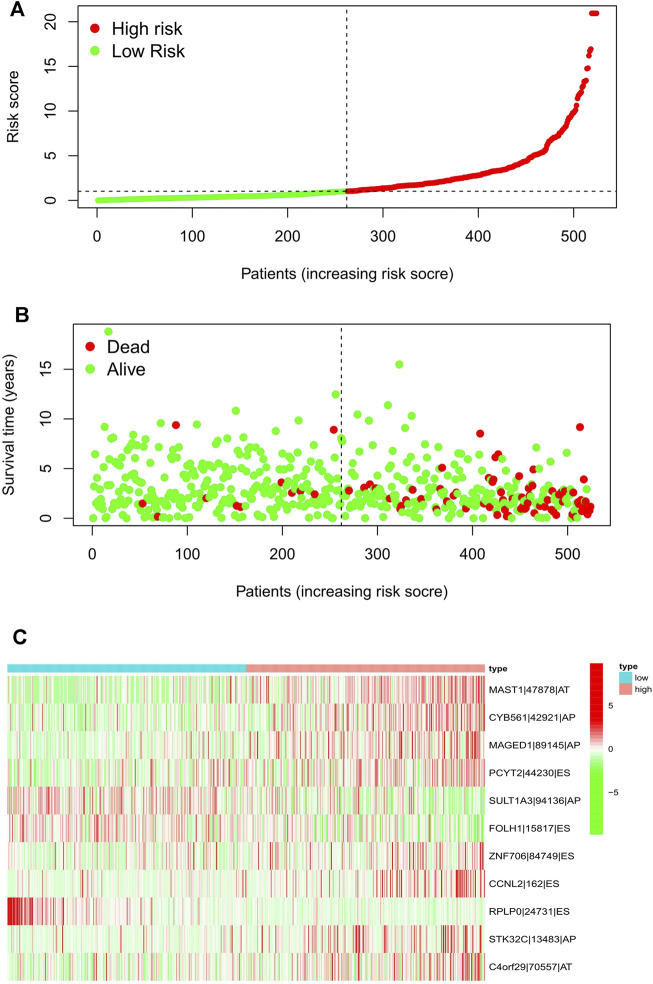
Risk score distribution of the 11-AS events signature in the TCGA cohort. Risk scores **(A)**, survival status **(B)**, and heatmap **(C)** of the 11-AS events PSI profiles were shown from top to bottom. AA, alternate acceptor; AD, alternate donor; AP, alternate promoter; AT, alternate terminator; ES, exon skip; ME, mutually-exclusive exons; RI, retained intron.

Meanwhile, the risk scores of each UCEC patient were calculated, and all patients were divided into low- and high-risk groups bound by the median risk score. The distribution diagram of survival risk score (Figure 2A), survival status of EC patients (Figure 2B), and clustering heatmap of the PSI levels of eleven-AS markers (Figure 2C) are shown. The horizontal axis displays the patients’ order of risk score from low to high (Figure 2).

### Construction and Evaluation of the Nomogram

**FIGURE 3:**
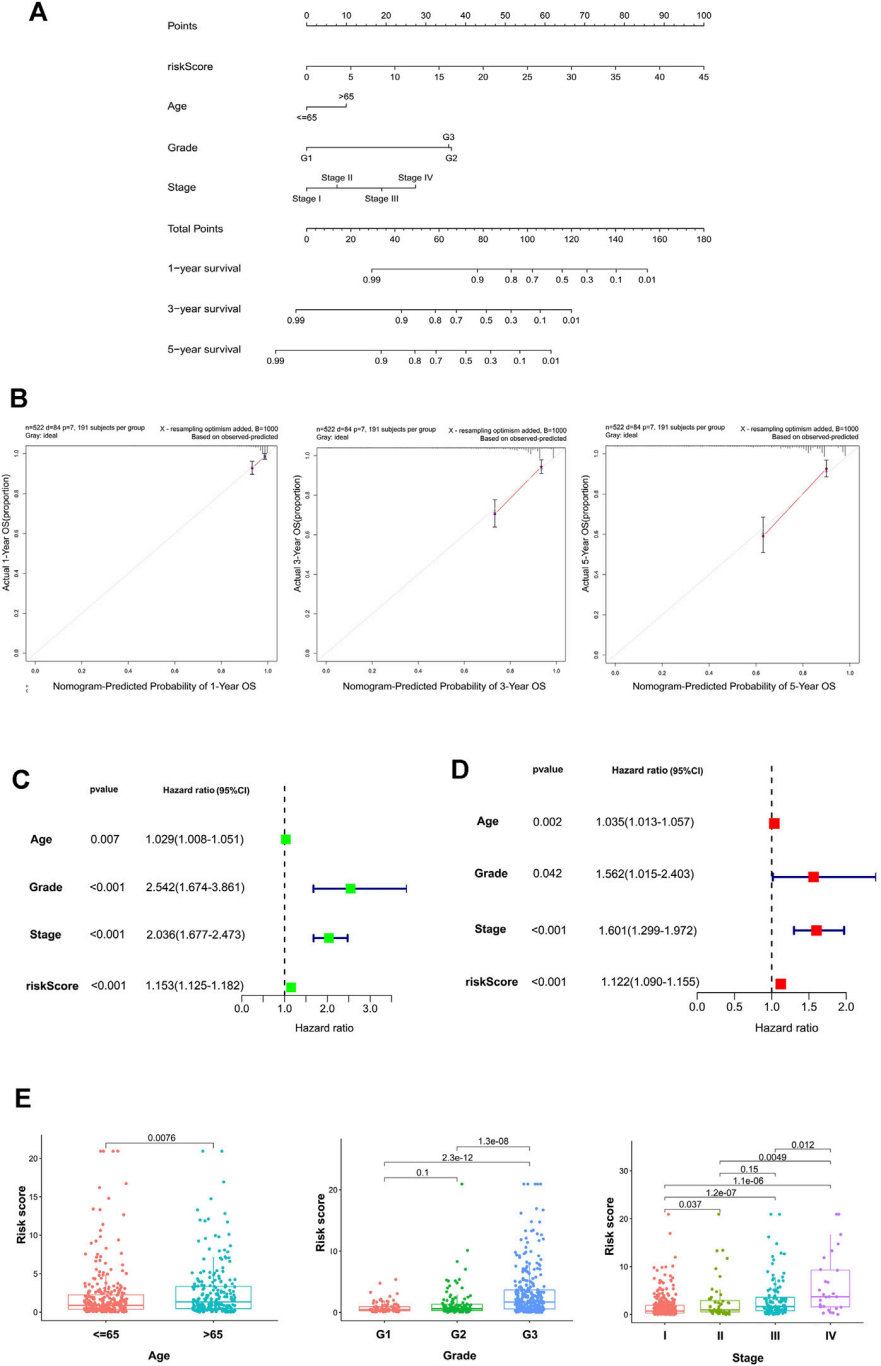
The establishment and validation of the nomogram. **(A)** The nomogram consisted of age, gender, FIGO stage, and risk score, and was used to predict the 1-, 3-, and 5-year survival probability of EC patients. **(B)** Calibration plots of the AS-clinical nomogram are in agreement between the nomogram-predicted and observed 1-, 3-, and 5-year outcomes of the UCEC cohort. The nomogram-predicted survival probability is plotted on the *x*-axis, and the actual survival is plotted on the *y*-axis. The 45° dashed line represents the ideal performance. The red lines represent the actual performances of the model, and the figures from left to right depict the 1-, 3-, and 5-year results. Univariate analysis **(C)** and multivariate analysis **(D)** of risk scores and clinical characteristics that were simultaneously associated with OS. **(E)** Differences in the risk score in terms of age, grade, and FIGO stage groups. The bottom and top of the boxes are the 25th and 75th percentiles (interquartile range).

The calibration curve demonstrated that the predicted values are satisfactorily consistent in the prediction of the 1-, 3-, and 5-year OS because the red lines in three pictures are almost overlap with the 45° dashed lines (Figure 3B). The box charts in Figure 3E show whether there are differences in patients’ risk score among different clinical index (age, grade, stage). The risk score of patients >65 years old was higher than that of patients ≤65 years old. With the increase of grade and stage of UCEC, the risk score increased gradually (Figure 3B).

Univariate and multivariate Cox regression methods were used and combined with patient clinical characteristics (age, grade, and FIGO stage) to analyze whether the 11-AS event signature could be an independent predictor of survival in patients with UCEC. When the *p* values of univariate analysis and multivariate analysis were both less than 0.05, it was considered that the model could be an independent prognostic factor. As depicted in Figures 3C,D, the results showed that the risk score could still be used as a reliable and stable independent risk predictor in the UCEC cohort (*p* < 0.001; Figures 3C,D). We then constructed a predictive nomogram based on the multivariate analysis (Figure 3A) that included risk scores and clinical characteristics. The results demonstrated that the risk score had satisfactory diagnostic ability and clinical characteristics (*p* < 0.05).

### The Risk Score and AS Events Are Associated With the Infiltration of Immune Cells in the UCEC Microenvironment

**FIGURE 4:**
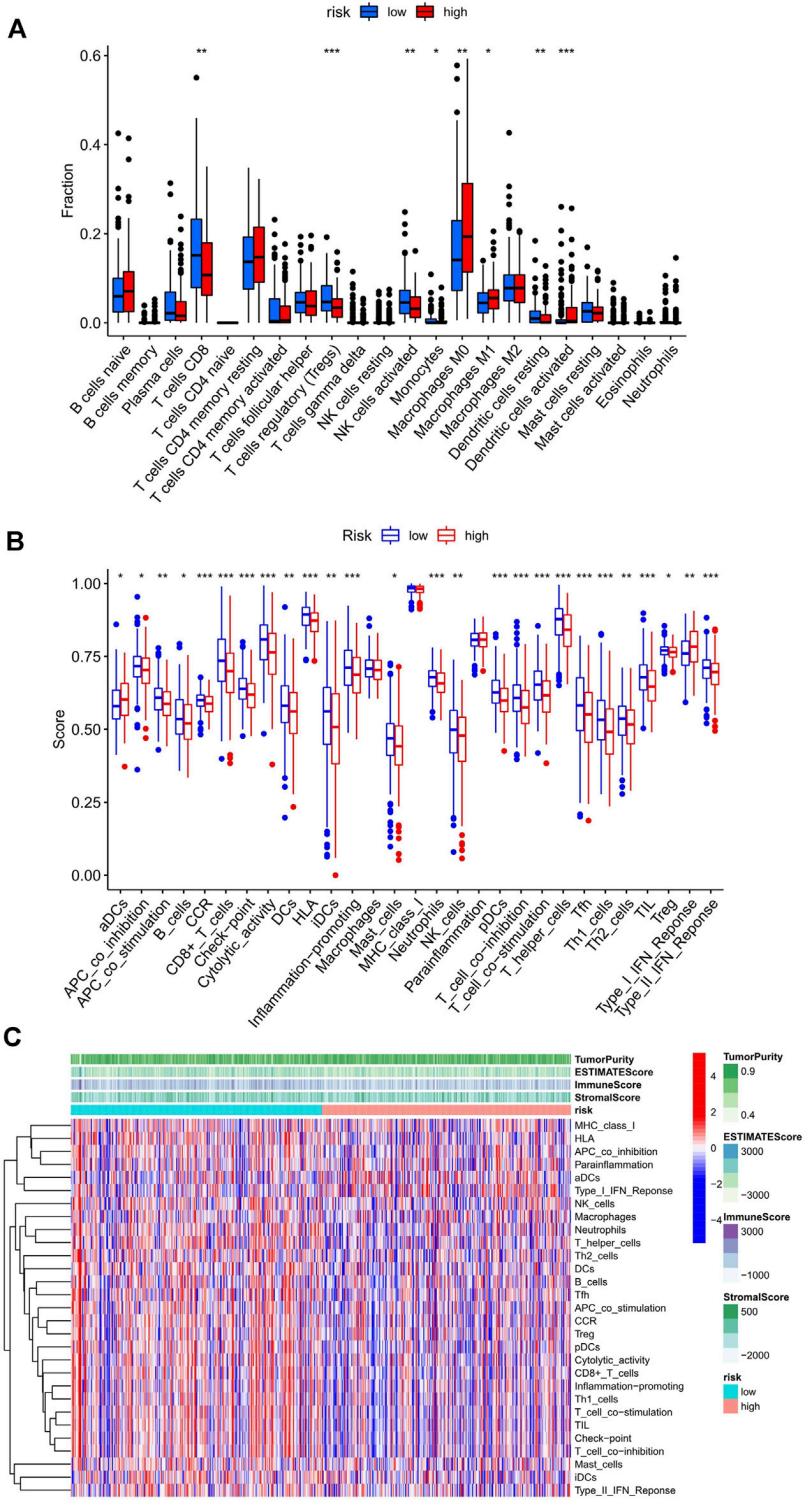
Relationship between the risk score and infiltrating immune cells in the UCEC tumor-immune microenvironment. **(A)** The landscape of 22 types of infiltrating immune cells in the low-risk score (*n* = 262) and high-risk score (*n* = 262) groups. **(B)** The landscape of 29 types of infiltrating immune cells and immune function in the two groups. The bottom and top of the boxes are the 25th and 75th percentiles (interquartile range). Blue: low risk, red: high risk. ***p < 0.001, **p < 0.01, *p < 0.05. **(C)** The heatmap showed a difference in the infiltrating immune cells between the two groups in the UCEC tumor-immune microenvironment.

First, the immune score in 29 types of infiltrating immune cells and immune function was assessed by the ssGSEA method (He et al., 2018). Figures 4B,C show the immune score differences of each immune cell in the low and high-risk score groups (Figures 4B,C). We further explored the impact of the risk score on the infiltration of 22 types of immune cells in the tumor microenvironment using the CIBERSORT algorithm. The landscape of 22 types of infiltrating immune cells in the low and high-risk score groups is shown in Figure 4A. Differential analysis results showed that eight types of immune cells [CD8 T cells, regulatory T cells (Tregs), activated natural killer (NK) cells, monocytes, M0 macrophages, M1 macrophages, resting dendritic cells, and activated dendritic cells] were significantly different between the two groups (*p* < 0.05).

### The Risk Score is Associated With the Key Immune Checkpoint Genes in the UCEC Tumor-Immune Microenvironment

**FIGURE 5:**
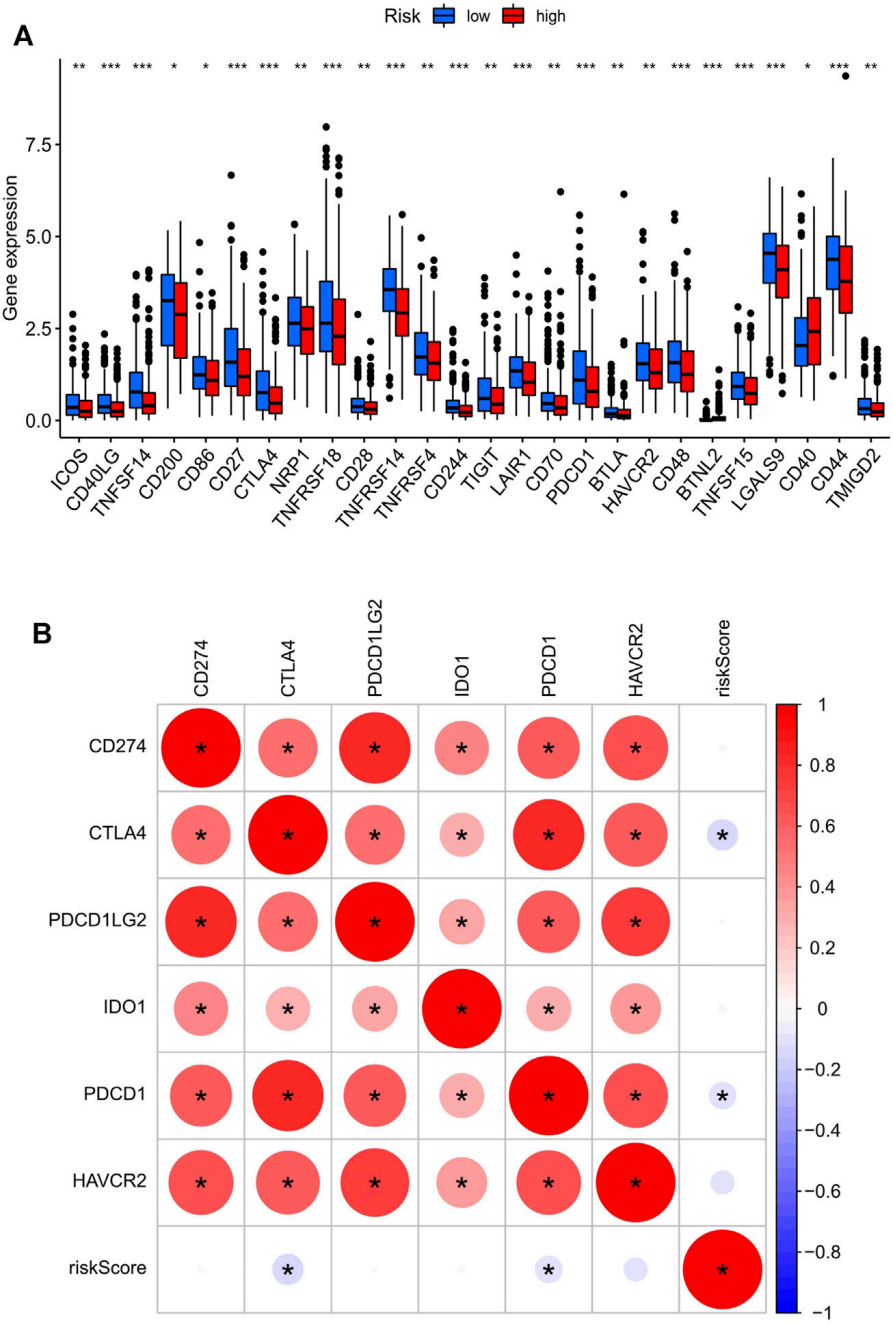
The key immune checkpoint genes are related to the risk score in the UCEC tumor-immune microenvironment. **(A)** The landscape of 26 types of immune checkpoint genes in low- and high-risk score groups. ***p < 0.001, **p < 0.01, *p < 0.05. **(B)** The correlation between the risk score and the six most important checkpoint genes (CD274, PDCD1, PDCD1LG2, CTLA4, HAVCR2, and IDO1). The bottom and top of the boxes are the 25th and 75th percentiles (interquartile range). *: statistically significant; red: positive correlation, blue: negative correlation.

The difference in the expression level of 47 immune checkpoint genes in the low- and high-risk score groups was assessed, and 26 genes were found to have significant differences (Figure 5A). Next, R packages (limma, corrplot, ggpubr, and ggExtra) were used to screen the risk scores related to the six most important checkpoint genes (CD274, PDCD1, PDCD1LG2, CTLA4, HAVCR2, and IDO1). Two immune checkpoint genes, PDCD1 and CTLA4, with negative correlation with risk score were identified (*p* < 0.001; Figure 5B). The scatter plot displaying the correlation of these two genes and the risk score were plotted separately. Although two of the correlation coefficients did not reach 0.3, the scatter plot showed a negative correlation (Supplementary Figure S1). At the same time, we can find that the expression of PDCD1 and CTLA4 in the high-risk score group was lower than that in the low-risk score group (Figure 5A).

### Extraction of IRGs Depending on AS Events and Their Correlation With Clinical Parameters

The expressions of 11 genes (Table 2) were identified to analyze the difference between UCEC and normal cohorts by the “limma” package (with the threshold of |log2FC| > 1 and *p* < 0.05), and two genes, CYB561 (|logFC| = 1.1892, *p* < 0.001) and FOLH1 (|logFC| = 1.0862, *p* < 0.001), were extracted for further analysis. Next, we divided the tumor patients into high- and low-expression groups according to the optimal cut-offs in CYB561 and FOLH1 (4.67 in CYB561, 2.64 in FOLH1) for clinical prognostic analysis.

**FIGURE 6:**
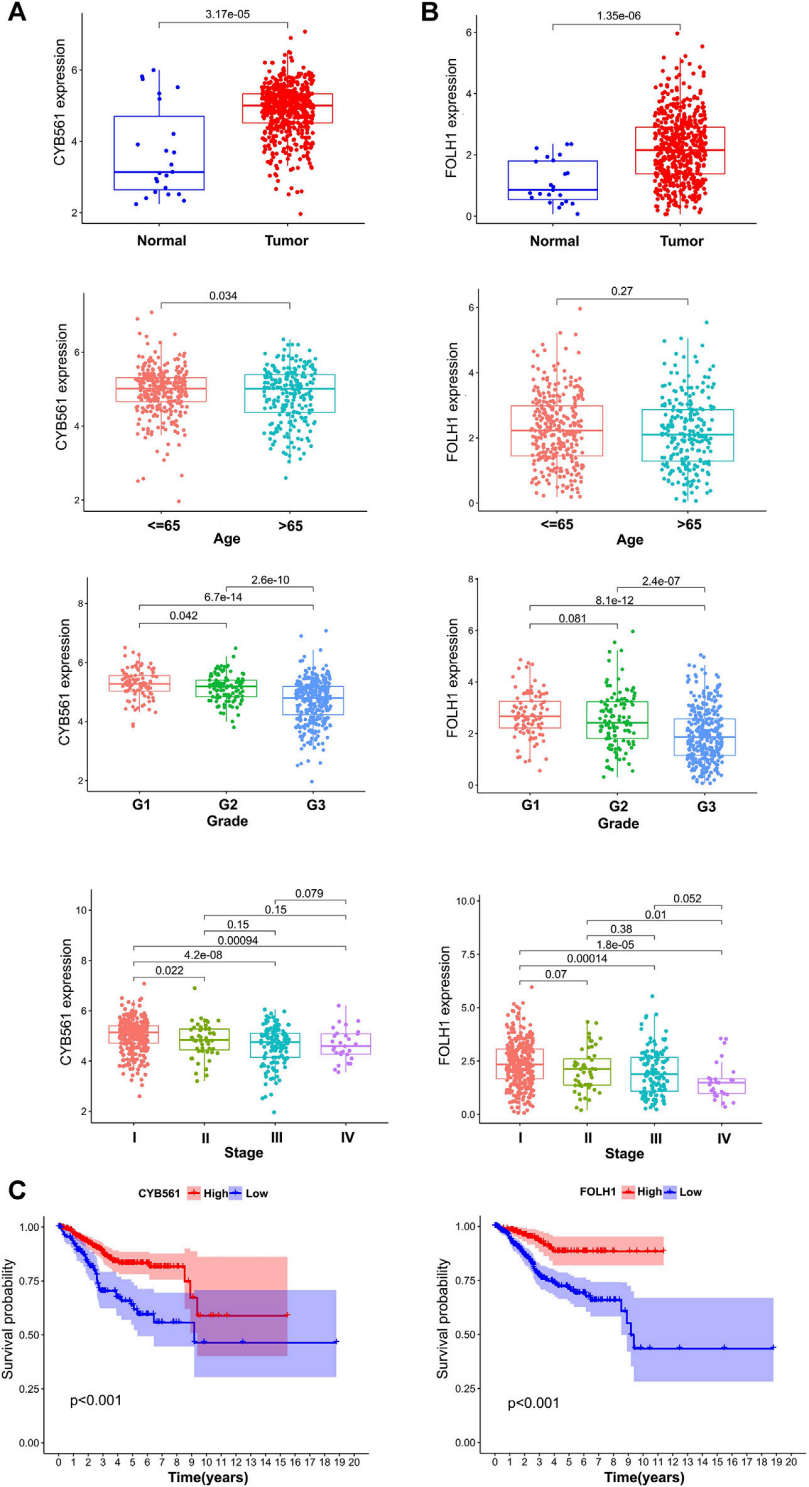
The prognostic signature of CYB561 and FOLH1 expression. The expression of CYB561 **(A)** and FOLH1 **(B)** in age, grade, and stage groups. **(C)** Kaplan-Meier survival curve of CYB561 and FOLH1 in high- and low-expression groups. The bottom and top of the boxes are the 25th and 75th percentiles (interquartile range).

The correlations between the expression of the two hub genes and the clinicopathological parameters were evaluated using R packages (limma, survival, and survminer). CYB561 and FOLH1 expression levels were significantly associated with grade and FIGO stage (*p* < 0.05). The expression of CYB561 and FOLH1 decreased gradually with increases in the grade and stage. However, no notable association between the two genes and age was observed (*p* ≥ 0.05) (Figures 6A,B). Survival analysis revealed that the low expressions of both CYB561 and FOLH1 were associated with poor prognosis (*p* < 0.001) (Figure 6C).

### Associations Between CYB561 Expression and Immune Cell Infiltration

**FIGURE 7:**
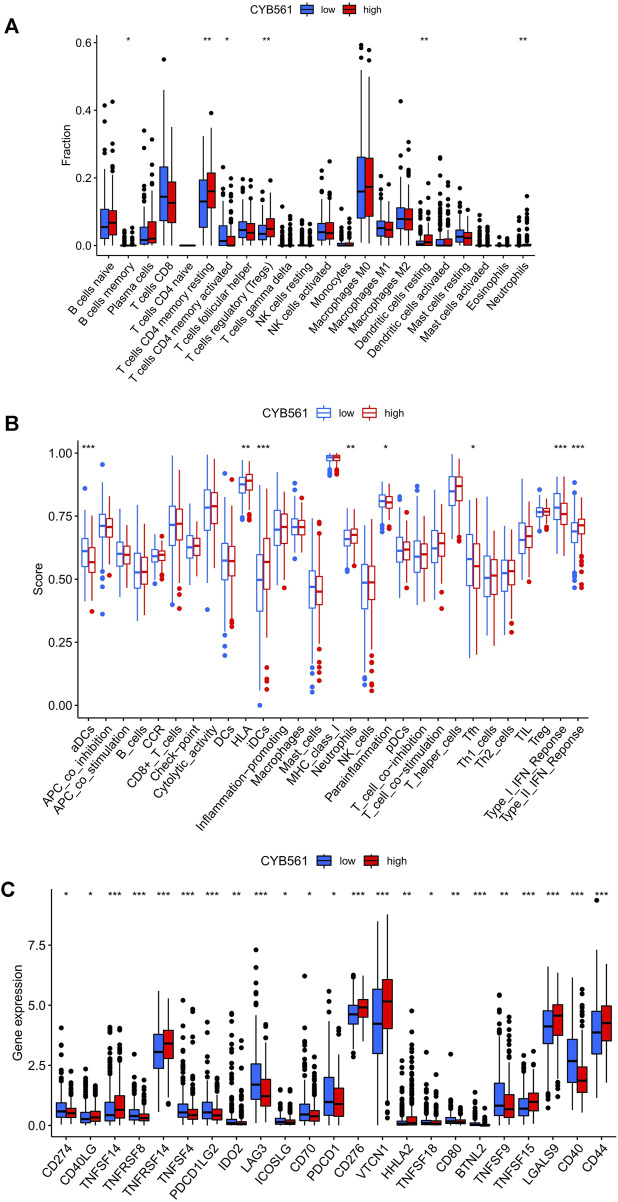
Relationship between CYB561 expression and infiltrating immune cells in the UCEC tumor-immune microenvironment. **(A)** The box plot shows the proportion difference of each immune cell between the low- and high- CYB561 expression groups. **(B)** The landscape of infiltrating immune cells and immune function in both groups. **(C)** The expression of CYB561 was associated with the key immune checkpoint genes in the UCEC microenvironment. The bottom and top of the boxes are the 25th and 75th percentiles (interquartile range). Blue: low risk, red: high risk. ***p < 0.001, **p < 0.01, *p < 0.05.

The landscape of 22 types of infiltrating immune cells in the low- and high- CYB561 expression groups are shown in Figure 7A. The two groups differed between resting dendritic cells, neutrophils, activated memory CD4 T cells, resting memory CD4 T cells, and Tregs. Figure 7B shows the immune score difference of each immune cell in the two groups (Figure 7B). We investigated the association between CYB561 expression and the tumor-infiltrating immune cells in UCEC using the TIMER database. The results demonstrated that CYB561 expression was positively correlated with B cell, CD4^+^ T cells, and CD8^+^ T cells, and was negatively correlated with myeloid dendritic cells, macrophages, and neutrophils (*p* < 0.05) (Supplementary Figure S2). However, we found no strong correlation between immune cell infiltration and CYB561 expression. Given that the risk score was related to tumor immunity, we finally appraised the correlation between the gene signature and the expression of immune checkpoints. Figure 7C shows the 23 immune checkpoints with differential expression in the low- and high-CYB561 expression groups. The gene expression of CD40LG, TNFSF14, TNFRSF14, CD276, VTCN1, HHLA2, TNFSF15, LGALS9, and CD44 was lower in the low-CYB561 expression groups (Figure 7C).

### Correlations Between FOLH1 Expression and Immune Cell Infiltration

**FIGURE 8:**
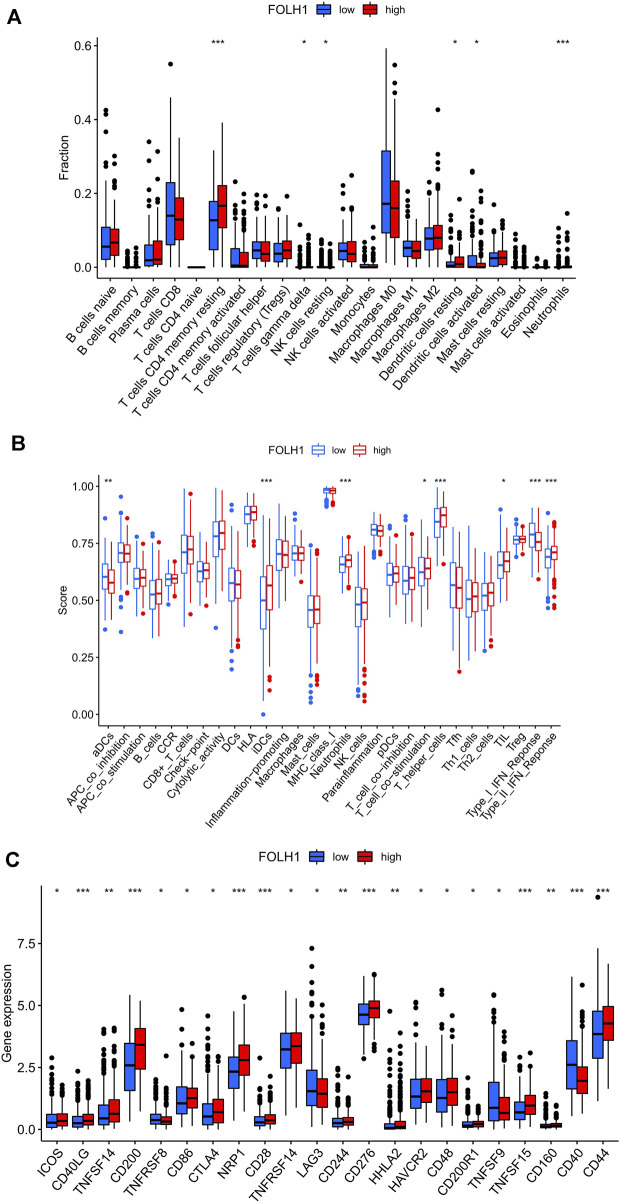
Relationship between FOLH1 expression and infiltrating immune cells in the UCEC tumor-immune microenvironment. **(A)** The box plot shows the proportion difference of each immune cell between the low- and high-FOLH1 expression groups. **(B)** The landscape of infiltrating immune cells and immune function in the two groups. **(C)** The expression of FOLH1 was associated with key immune checkpoint genes in the tumor microenvironment (TME). The bottom and top of the boxes are the 25th and 75th percentiles (interquartile range). Blue: low risk, red: high risk. ***p < 0.001, **p < 0.01, *p < 0.05.

The landscape of 22 types of infiltrating immune cells in the low- and high- FOLH1 expression groups are shown in Figure 8A. Resting memory CD4 T cells, gamma delta T cells, resting NK cells, resting dendritic cells, activated dendritic cells, and neutrophils were different in the two groups. Figure 8B shows the immune score difference of each immune cell in the two groups. We further investigated the association between FOLH1 expression and the tumor-infiltrating immune cells in UCEC. The results showed that FOLH1 expression was positively correlated with macrophages, CD4^+^ T cells, and CD8^+^ T cells, and was negatively correlated with B cells, myeloid dendritic cells, and neutrophils (*p* < 0.05) (Supplementary Figure S3). However, we also found no strong correlation between immune cell infiltration and FOLH1 expression. Figure 8C shows the immune checkpoints with differential expression in the low- and high-FOLH1 expression groups.

### The Tumor Purity, ESTIMATE Score, Immune Score, Stromal Score and Between the Low- and High-Expression Groups of CYB561 and FOLH1

**FIGURE 9:**
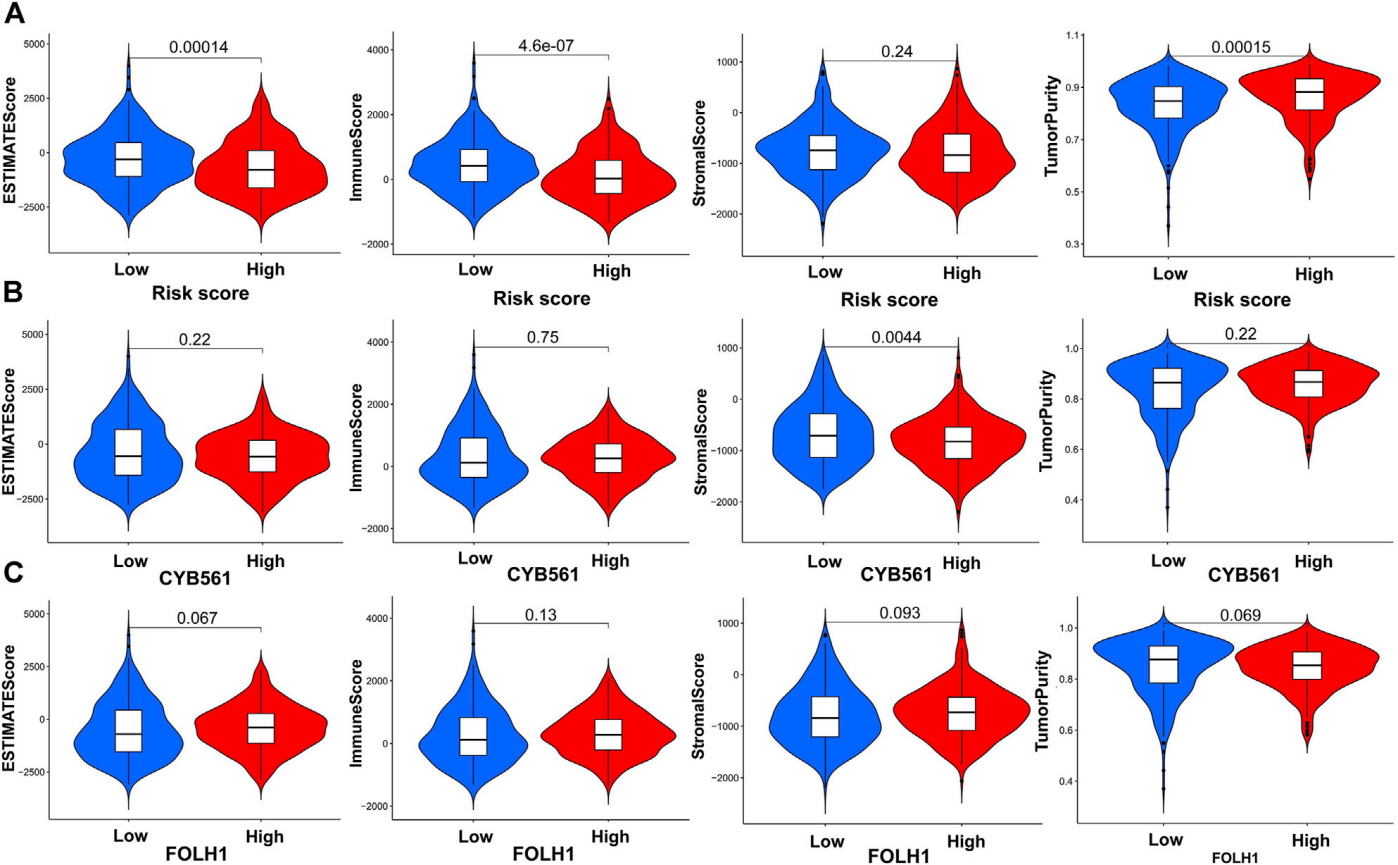
Construction and verification of the immune-related UCEC subgroups. **(A)** The violin plot shows the differences in Tumor Purity, ESTIMATE Score, Immune Score, and Stromal Score between the high- and low-risk score groups, calculated using the ESTIMATE algorithm. Comparison of Stromal Scores, Immune Scores, ESTIMATE Scores, and Tumor Purity between two groups of CYB561 **(B)** and FOLH1**(C)**.

First, the violin plot assessed the differences in Tumor Purity, ESTIMATE Score, Immune Score, and Stromal Score between the two groups, calculated using the ESTIMATE algorithm (Figure 9A). ESTIMATE Score and Immune Score were higher in the low-risk score group, while Tumor Purity in the low-risk score group was lower than that in the high-risk score group (*p* < 0.05). The Stromal Score showed no difference (*p* ≥ 0.05).

In order to determine the effectiveness of the grouping strategy between the low- and high-expression groups of CYB561 and FOLH1, the ESTIMATE method was applied to evaluate Tumor Purity, ESTIMATE Score, Immune Score, and Stromal Score. Compared with the high-CYB561 expression group, the low expression group had a higher Stromal Score (*p* < 0.05) (Figure 9B). The other parameters had no differences between the two groups in CYB561 and FOLH1 (*p* ≥ 0.05) (Figures 9B,C).

### The Quantity of Six Immune Cells in Gene Copy Number of CYB561 and FOLH1

The correlations between the CYB561 copy number and six immune cells were also analyzed using the TIMER database. The number of cells was found to decrease with the increase in the gene copy number in myeloid DC cells and CD8^+^ T cells, and was found to decrease with the decrease in the gene copy number in myeloid DC cells and CD8^+^ T cells (*p* < 0.05) (Supplementary Figure S4). Next, we analyzed the correlations between the FOLH1 copy number and six immune cells. The number of cells was found to decrease with the increase in the gene copy number in myeloid DC cells, macrophage, CD8^+^ T cells, and the cells number was found to decrease with the reduce in the gene copy number in myeloid DC cells and CD8^+^ T cells (*p* < 0.05) (Supplementary Figure S5). Notably, we observed that the change in gene copy numbers in the two hub genes led to the deletion number of both myeloid DC cells and CD8^+^ T cells.

### The Potential Regulatory Network Between SFs and AS Events

**FIGURE 10:**
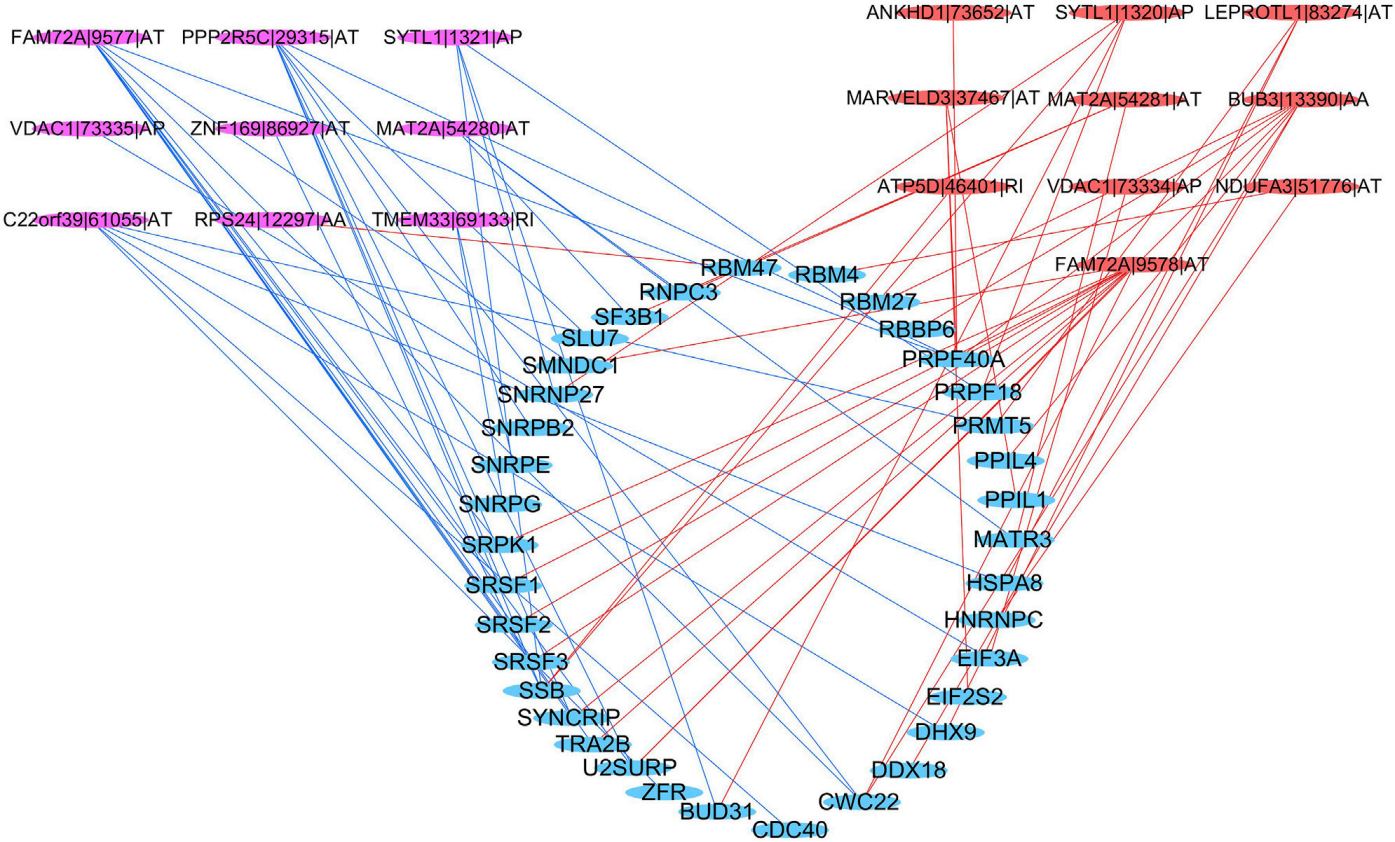
Regulatory network between SFs and AS events. Blue ellipse: spliced factors, purple ellipse: low-risk AS events, red ellipse: high-risk AS events, blue line: negative correlation, red line: a positive correlation.

Thirty-six SFs (blue) were found to be significantly related to 19 survival-associated AS events, consisting of nine low-risk AS events (RPS24|12297|AA, ZNF169|86927|AT, MAT2A|54280|AT, C22orf39|61055|AT, VDAC1|73335|AP, FAM72A|9577|AT, PPP2R5C|29315|AT, TMEM33|69133|RI, SYTL1|1321|AP; purple) and 10 high-risk AS events (LEPROTL1|83274|AT, ANKHD1|73652|AT, MAT2A|54281|AT, BUB3|13390|AA, ATP5D|46401|RI, FAM72A|9578|AT, VDAC1|73334|AP, MARVELD3|37467|AT, NDUFA3|51776|AT,SYTL1|1320|AP; red). The majority of low-risk AS events were negatively correlated with SF expression (blue lines), and all of the high-risk AS events were positively correlated with SF expression (red lines) (Figure 10).

## Discussion

Dysregulation of AS can affect essential biological processes and thus drive disease-associated pathophysiology (Gamazon and Stranger, 2014). Emerging data have demonstrated that aberrant AS events are closely associated with cancer progression, metastasis, therapeutic resistance, and other oncogenic processes (Climente-González et al., 2017). Cancer cells have general and cancer type-specific and subtype-specific alterations in the splicing process, which can have prognostic value and contribute to every hallmark of cancer progression, including the cancer-immune responses (Bonnal et al., 2020). Moreover, substantial preclinical work has identified a variety of small molecule compounds and genetic and other approaches to target the spliceosome or its products with potential therapeutic effects (Bonnal et al., 2020). Therefore, it is of great importance to further study the characteristics of AS in the immune microenvironment for UCEC immunotherapy. In recent years, the relationship between AS and UCEC has been studied. In endometrial cancer, AS of vascular endothelial growth factor A (VEGF-A) is regulated by RBM10 (Dou et al., 2020a). Popli et al. found that SF3B1 plays a crucial oncogenic role in the tumorigenesis of EC and hence may support the development of SF3B1 inhibitors to treat this disease (Dou et al., 2020a). XQ et al. showed that miR-335 modulates Numb AS *via* targeting RBM10 in EC (Dou et al., 2020b).

We extracted IRGs depending on AS events and examined their correlation with clinical parameters. Finally, two AS-related genes, CYB561|42921|AP and FOLH1|15817|ES, were extracted from the 11 genes involved in the AS prognostic model. CYB561 encodes the protein CYB561, named as such because of its optical absorbance at 561 nm CYB561 is a heme-containing enzyme that is necessary for the continuous regeneration of semidehydroascorbate to ascorbate inside chromaffin granules and neuropeptide secretory vesicles (van den Berg et al., 2018). It is widely expressed in the adrenal glands, prostate, and 23 other tissues, including the endometrium. However, data on the role of the CYB561 gene in human cancers are very limited. A meta-analysis showed that low mRNA expression of CYB561 was prognostic of a poor outcome in ovarian cancer (Willis et al., 2016). CYB561 serves as a potential prognostic biomarker and target for breast cancer (Yang et al., 2021). We found the expression of CYB561 decreased gradually with the increased grade and FIGO stage which indicated a lower survival probability (*p* < 0.001) in UCEC. Our results were consistent with the previous ones. Besides, alternate promoter of CYB561 was associated with the OS of UCEC patients (*p* = 0.0003) in our research. The changes in transcription is regarded as a defining feature of cancer. Most human protein-coding genes are regulated by multiple, distinct promoters, suggesting that the selection of promoter is closely related to the expression of target gene (Demircioğlu et al., 2019). How the AP contributes the low expression of CYB561 in endometrial carcinoma remains to be further explored.

FOLH1 is also known as prostate-specific membrane antigen (PSMA), which encodes a transmembrane glycoprotein that acts as a glutamate carboxypeptidase on different alternative substrates. In the prostate, this protein is up-regulated in cancerous cells and is used as an effective diagnostic and prognostic indicator of prostate cancer (Date et al., 2017). PSMA is highly and specifically expressed in the neovasculature of ovarian, endometrial, and cervical squamous carcinomas (Wernicke et al., 2017). Mhawech-Fauceglia et al. showed that PSMA is under-expressed in advanced stage endometrial adenocarcinoma (Mhawech-Fauceglia et al., 2008), which is consistent with our findings. Their research indicated that the loss of PSMA expression can be considered a prognostic marker in patients with endometrial adenocarcinoma and could be due to epigenetic silencing (Mhawech-Fauceglia et al., 2008). FOLH1 likely arose from a duplication event of a nearby chromosomal region. Alternative splicing gives rise to multiple transcript variants encoding several different isoforms (Watt et al., 2001; Zink et al., 2020). Our research found ES in FOLH1 was associated with the OS of UCEC patients (*p* = 0.0000). What’s more, ES was also the most frequent splice type among the seven AS types (34.4%) in UCEC. If the normal exon can be restored into the exon of ES occurred, it will bring hope to the treatment of many diseases (Verhaart and Aartsma-Rus, 2019). However, the mechanism of ES in FOLH1 leading to a high stage and poor prognosis of UCEC is unknown.

Next, two immune checkpoint genes, Cytotoxic Lymphocyte Antigen 4 (CTLA-4) and Programmed Cell Death 1 (PDCD1), showed negative correlations with the risk score of AS in UCEC. CTLA-4 is expressed on the surface of naive effector T cells and Tregs (Billeskov et al., 2017; Menéndez-Menéndez et al., 2019). Based on its role as a negative regulator of T cell activation, CTLA-4 has become an attractive target for therapies aiming to enhance the effector activity of T lymphocytes. The first targeted drug for CTLA-4, ipilimumab, was approved by the Food and Drug Administration (FDA) in 2011 to treat melanoma (Lipson and Drake, 2011). At present, both nivolumab and ipilimumab are undergoing phase II clinical trials in UCEC (Grywalska et al., 2019). In our study, the CTLA-4 gene expression, the number and immune score of Tregs all decreased in the high-risk score group, which predicted a worse prognosis. Therefore, we supposed that a high-risk score of AS might be related to the decreased immune activity of Treg cells and the low expression of CTLA-4. It is possible that the targeted regulation of AS can improve the immune activity of Treg cells and increase the expression of CTLA-4, which may be valuable in improving the survival rate of UCEC patients, although further confirmation is needed.

PDCD1, also known as PD-1, functions primarily in peripheral tissues. It is expressed on the surface of activated T cells, Tregs, activated B cells, and NK cells (Page et al., 2014). In 2014, the first FDA-approved immune checkpoint inhibitor targeting PD-1 was nivolumab (Grywalska et al., 2019). During the 2015 annual meeting of the Society of Gynecologic Oncology, Herzog et al. reported that the highest PD-1 expression rates among studied cancer types were in EC (75.2%) (Page et al., 2014). We found that PDCD1 expression was suppressed in the high-risk score group, and the 5-year survival rate was lower than that in the low-risk score group. It has been confirmed that there are variable splicing events in the PD1 gene (Nielsen et al., 2005; Wang et al., 2021). Another research considered the AS events in PD-1 may be a novel source for diagnostic and therapeutic target on celiac disease (Ponce de León et al., 2021). Why the high-risk AS events in PDCD1 lead to a worse prognosis of endometrial cancer needs further study.

The current study also has several limitations that should be noted. Firstly, this study is based on bioinformatics analysis, and there are no recruited cohorts for prognostic verification. Secondly, the values of the two-gene signatures for immunotherapeutic drugs prediction have not been verified in patient cohorts.

## Conclusion

This study assessed the heterogeneity of tumor-infiltrating immune cells in UCEC and identified two AS-related genes, CYB561 and FOLH1, from the 11 genes involved in the AS prognostic model. Two immune checkpoint genes, CTLA4 and PDCD1, were negatively correlated with the risk score. The outcomes of this study are significant for investigating the immune-related mechanisms of tumor progression and exploring novel prognostic predictors and precise therapy methods.

## Data Availability

The datasets presented in this study can be found in online repositories. The names of the repository/repositories and accession number(s) can be found in the article/Supplementary Material.

## Supplementary Material

Supplementary Figure S1 The correlation between the risk score and the checkpoint genes PDCD1 and CTLA4.

Supplementary Figure S2 The association between CYB561 expression and the tumor-infiltrating immune cells in UCEC.

Supplementary Figure S3 The association between FOLH1 expression and the tumor-infiltrating immune cells in UCEC.

Supplementary Figure S4 Relationship between the gene copy number of CYB561 and the quantity of six immune cells using the TIMER database.

Supplementary Figure S5 Relationship between the gene copy number of FOLH1 and the quantity of six immune cells using the TIMER database.

## References

[B1] Baralle F. E. Giudice J. (2017). Alternative Splicing as a Regulator of Development and Tissue Identity. Nat. Rev. Mol. Cell. Biol. 18, 437–451. 10.1038/nrm.2017.27 28488700 PMC6839889

[B2] Billeskov R. Wang Y. Solaymani-Mohammadi S. Frey B. Kulkarni S. Andersen P. (2017). Low Antigen Dose in Adjuvant-Based Vaccination Selectively Induces CD4 T Cells with Enhanced Functional Avidity and Protective Efficacy. J. Immunol. 198 (9), 3494–3506. 10.4049/jimmunol.1600965 28348274 PMC5392729

[B3] Bonnal S. C. López-Oreja I. Valcárcel J. (2020). Roles and Mechanisms of Alternative Splicing in Cancer - Implications for Care. Nat. Rev. Clin. Oncol. 17 (8), 457–474. 10.1038/s41571-020-0350-x 32303702

[B4] Bray F. Ferlay J. Soerjomataram I. Siegel R. L. Torre L. A. Jemal A. (2018). Global Cancer Statistics 2018: GLOBOCAN Estimates of Incidence and Mortality Worldwide for 36 Cancers in 185 Countries. CA A Cancer J. Clin. 68 (6), 394–424. 10.3322/caac.21492 30207593

[B5] Buratti E. Baralle M. Baralle F. E. (2006). Defective Splicing, Disease and Therapy: Searching for Master Checkpoints in Exon Definition. Nucleic Acids Res. 34 (12), 3494–3510. 10.1093/nar/gkl498 16855287 PMC1524908

[B6] Climente-González H. Porta-Pardo E. Godzik A. Eyras E. (2017). The Functional Impact of Alternative Splicing in Cancer. Cell Rep. 20 (9), 2215–2226. 10.1016/j.celrep.2017.08.012 28854369

[B7] Couzin-Frankel J. (2013). Breakthrough of the Year 2013. Cancer Immunotherapy. Science 342 (6165), 1432–1433. 10.1126/science.342.6165.1432 24357284

[B8] Date A. A. Rais R. Babu T. Ortiz J. Kanvinde P. Thomas A. G. (2017). Local Enema Treatment to Inhibit FOLH1/GCPII as a Novel Therapy for Inflammatory Bowel Disease. J. Control. Release 263, 132–138. 10.1016/j.jconrel.2017.01.036 28159515 PMC5661937

[B9] de Necochea-Campion R. Shouse G. P. Zhou Q. Mirshahidi S. Chen C.-S. (2016). Aberrant Splicing and Drug Resistance in AML. J. Hematol. Oncol. 9 (1), 85. 10.1186/s13045-016-0315-9 27613060 PMC5018179

[B10] Demircioğlu D. Cukuroglu E. Kindermans M. Nandi T. Calabrese C. Fonseca N. A. (2019). A Pan-Cancer Transcriptome Analysis Reveals Pervasive Regulation through Alternative Promoters. Cell 178 (6), 1465–1477.e17. 10.1016/j.cell.2019.08.018 31491388

[B11] Dou X. Q. Chen X. J. Wen M. X. Zhang S. Z. Zhou Q. Zhang S. Q. (2020). Alternative Splicing of VEGFA Is Regulated by RBM10 in Endometrial Cancer. Kaohsiung J. Med. Sci. 36 (1), 13–19. 10.1002/kjm2.12127 31587503 PMC11896373

[B12] Dou X. Q. Chen X. J. Zhou Q. Wen M. X. Zhang S. Z. Zhang S. Q. (2020). miR‐335 Modulates Numb Alternative Splicing via Targeting RBM10 in Endometrial Cancer. Kaohsiung J. Med. Sci. 36 (3), 171–177. 10.1002/kjm2.12149 31894898 PMC11896503

[B13] Dvinge H. Kim E. Abdel-Wahab O. Bradley R. K. (2016). RNA Splicing Factors as Oncoproteins and Tumour Suppressors. Nat. Rev. Cancer 16 (7), 413–430. 10.1038/nrc.2016.51 27282250 PMC5094465

[B14] Frankiw L. Baltimore D. Li G. (2019). Alternative mRNA Splicing in Cancer Immunotherapy. Nat. Rev. Immunol. 19 (11), 675–687. 10.1038/s41577-019-0195-7 31363190

[B15] Gamazon E. R. Stranger B. E. (2014). Genomics of Alternative Splicing: Evolution, Development and Pathophysiology. Hum. Genet. 133 (6), 679–687. 10.1007/s00439-013-1411-3 24378600

[B16] Gilbert W. (1978). Why Genes in Pieces? Nature 271 (5645), 271501–501. 10.1038/271501a0 622185

[B17] Grywalska E. Sobstyl M. Putowski L. Roliński J. (2019). Current Possibilities of Gynecologic Cancer Treatment with the Use of Immune Checkpoint Inhibitors. Int. J. Mol. Sci. 20 (19), 4705. 10.3390/ijms20194705 PMC6801535 31547532

[B18] He Y. Jiang Z. Chen C. Wang X. (2018). Classification of Triple-Negative Breast Cancers Based on Immunogenomic Profiling. J. Exp. Clin. Cancer Res. 37 (1), 327. 10.1186/s13046-018-1002-1 30594216 PMC6310928

[B19] Heagerty P. J. Lumley T. Pepe M. S. (2000). Time-dependent ROC Curves for Censored Survival Data and a Diagnostic Marker. Biometrics 56 (2), 337–344. 10.1111/j.0006-341x.2000.00337.x 10877287

[B20] Kahles A. Lehmann K. V. Toussaint N. C. Hüser M. Stark S. G. Sachsenberg T. (2018). Comprehensive Analysis of Alternative Splicing across Tumors from 8,705 Patients. Cancer Cell 34, 211–224. 10.1158/0008-5472.can-04-1910 30078747 PMC9844097

[B21] Li H. Liu J. Shen S. Dai D. Cheng S. Dong X. (2020). Pan-cancer Analysis of Alternative Splicing Regulator Heterogeneous Nuclear Ribonucleoproteins (hnRNPs) Family and Their Prognostic Potential. J. Cell. Mol. Med. 24 (19), 11111–11119. 10.1111/jcmm.15558 32915499 PMC7576281

[B22] Lipson E. J. Drake C. G. (2011). Ipilimumab: an Anti-CTLA-4 Antibody for Metastatic Melanoma. Clin. Cancer Res. 17 (22), 6958–6962. 10.1158/1078-0432.CCR-11-1595 21900389 PMC3575079

[B23] Liu X. Liu C. Liu J. Song Y. Wang S. Wu M. (2021). Identification of Tumor Microenvironment-Related Alternative Splicing Events to Predict the Prognosis of Endometrial Cancer. Front. Oncol. 11, 645912. 10.3389/fonc.2021.645912 33996564 PMC8116885

[B24] Menéndez-Menéndez J. Hermida-Prado F. Granda-Díaz R. González A. García-Pedrero J. M. Del-Río-Ibisate N. (2019). Deciphering the Molecular Basis of Melatonin Protective Effects on Breast Cells Treated with Doxorubicin: TWIST1 a Transcription Factor Involved in EMT and Metastasis, a Novel Target of Melatonin. Cancers 11 (7), 1011. 10.3390/cancers11071011 PMC6679136 31331001

[B25] Mhawech-Fauceglia P. Smiraglia D. J. Bshara W. Andrews C. Schwaller J. South S. (2008). Prostate-specific Membrane Antigen Expression Is a Potential Prognostic Marker in Endometrial Adenocarcinoma. Cancer Epidemiol. Biomarkers Prev. 17 (3), 571–577. 10.1158/1055-9965.EPI-07-0511 18349274

[B26] Morice P. Leary A. Creutzberg C. Abu-Rustum N. Darai E. (2016). Endometrial Cancer. Lancet 387 (10023), 1094–1108. 10.1016/S0140-6736(15)00130-0 26354523

[B27] Newman A. M. Liu C. L. Green M. R. Gentles A. J. Feng W. Xu Y. (2015). Robust Enumeration of Cell Subsets from Tissue Expression Profiles. Nat. Methods 12 (5), 453–457. 10.1038/nmeth.3337 25822800 PMC4739640

[B28] Nielsen C. Ohm-Laursen L. Barington T. Husby S. Lillevang S. T. (2005). Alternative Splice Variants of the Human PD-1 Gene. Cell. Immunol. 235 (2), 109–116. 10.1016/j.cellimm.2005.07.007 16171790

[B29] Obeng E. A. Stewart C. Abdel-Wahab O. (2019). Altered RNA Processing in Cancer Pathogenesis and Therapy. Cancer Discov. 9 (11), 1493–1510. 10.1158/2159-8290.cd-19-0399 31611195 PMC6825565

[B30] Page D. B. Postow M. A. Callahan M. K. Allison J. P. Wolchok J. D. (2014). Immune Modulation in Cancer with Antibodies. Annu. Rev. Med. 65, 185–202. 10.1146/annurev-med-092012-112807 24188664

[B31] Ponce de León C. Lorite P. López-Casado M. Á. Barro F. Palomeque T. Torres M. I. (2021). Significance of PD1 Alternative Splicing in Celiac Disease as a Novel Source for Diagnostic and Therapeutic Target. Front. Immunol. 12, 678400. 10.3389/fimmu.2021.678400 34220824 PMC8242946

[B32] Popli P. Richters M. M. Chadchan S. B. Kim T. H. Tycksen E. Griffith O. (2020). Splicing Factor SF3B1 Promotes Endometrial Cancer Progression via Regulating KSR2 RNA Maturation. Cell Death Dis. 11 (10), 842. 10.1038/s41419-020-03055-y 33040078 PMC7548007

[B33] Ryan M. Wong W. C. Brown R. Akbani R. Su X. Broom B. (2016). TCGASpliceSeq a Compendium of Alternative mRNA Splicing in Cancer. Nucleic Acids Res. 44 (D1), D1018–D1022. 10.1093/nar/gkv1288 26602693 PMC4702910

[B34] van den Berg M. P. Almomani R. Biaggioni I. van Faassen M. van der Harst P. Silljé H. H. W. (2018). Mutations in CYB561 Causing a Novel Orthostatic Hypotension Syndrome. Circ. Res. 122 (6), 846–854. 10.1161/CIRCRESAHA.117.311949 29343526 PMC5924476

[B35] Venables J. P. (2004). Aberrant and Alternative Splicing in Cancer. Cancer Res. 64, 7647–7654. 10.1093/jmcb/mjz033 15520162

[B36] Verhaart I. E. C. Aartsma-Rus A. (2019). Therapeutic Developments for Duchenne Muscular Dystrophy. Nat. Rev. Neurol. 15 (7), 373–386. 10.1038/s41582-019-0203-3 31147635

[B37] Wang B.-D. Lee N. (2018). Aberrant RNA Splicing in Cancer and Drug Resistance. Cancers 10 (11), 458. 10.3390/cancers10110458 PMC6266310 30463359

[B38] Wang C. Weng M. Xia S. Zhang M. Chen C. Tang J. (2021). Distinct Roles of Programmed Death Ligand 1 Alternative Splicing Isoforms in Colorectal Cancer. Cancer Sci. 112 (1), 178–193. 10.1111/cas.14690 33058325 PMC7780007

[B39] Wang C. Zheng M. Wang S. Nie X. Guo Q. Gao L. (2019). Whole Genome Analysis and Prognostic Model Construction Based on Alternative Splicing Events in Endometrial Cancer. BioMed Res. Int. 2019, 2686875. 10.1155/2019/2686875 31355251 PMC6634061

[B40] Wang Q. Xu T. Tong Y. Wu J. Zhu W. Lu Z. (2019). Prognostic Potential of Alternative Splicing Markers in Endometrial Cancer. Mol. Ther. - Nucleic Acids 18, 1039–1048. 10.1016/j.omtn.2019.10.027 31785579 PMC6889075

[B41] Watt F. Martorana A. Brookes D. E. Ho T. Kingsley E. O'Keefe D. S. (2001). A Tissue-Specific Enhancer of the Prostate-Specific Membrane Antigen Gene, FOLH1. Genomics 73 (3), 243–254. 10.1006/geno.2000.6446 11350116

[B42] Wernicke A. G. Kim S. Liu H. Bander N. H. Pirog E. C. (2017). Prostate-specific Membrane Antigen (PSMA) Expression in the Neovasculature of Gynecologic Malignancies: Implications for PSMA-Targeted Therapy. Appl. Immunohistochem. Mol. Morphol. 25 (4), 271–276. 10.1097/PAI.0000000000000297 26862945

[B43] Willis S. Villalobos V. M. Gevaert O. Abramovitz M. Williams C. Sikic B. I. (2016). Single Gene Prognostic Biomarkers in Ovarian Cancer: A Meta-Analysis. PLoS One 11 (2), e0149183. 10.1371/journal.pone.0149183 26886260 PMC4757072

[B44] Yang X. Zhao Y. Shao Q. Jiang G. (2021). Cytochrome B561 Serves as a Potential Prognostic Biomarker and Target for Breast Cancer. Int. J. Gen. Med. 14, 10447–10464. 10.2147/IJGM.S338878 35002301 PMC8722309

[B45] Yoshihara K. Shahmoradgoli M. Martínez E. Vegesna R. Kim H. Torres-Garcia W. (2013). Inferring Tumour Purity and Stromal and Immune Cell Admixture from Expression Data. Nat. Commun. 4, 2612. 10.1038/ncomms3612 24113773 PMC3826632

[B46] Zink C. F. Barker P. B. Sawa A. Weinberger D. R. Wang M. Quillian H. (2020). Association of Missense Mutation in FOLH1 with Decreased NAAG Levels and Impaired Working Memory Circuitry and Cognition. Am. J. Psychiatry 177 (12), 1129–1139. 10.1176/appi.ajp.2020.19111152 33256444

