# Supplementary figures and images for: Identification of Alternative Splicing-Related Genes CYB561 and FOLH1 in the Tumor-Immune Microenvironment for Endometrial Cancer Based on TCGA Data Analysis
Source: Front Genet. 2022 Jun 28;13:770569. doi: 10.3389/fgene.2022.770569 (PMC9274141; doi:10.3389/fgene.2022.770569)

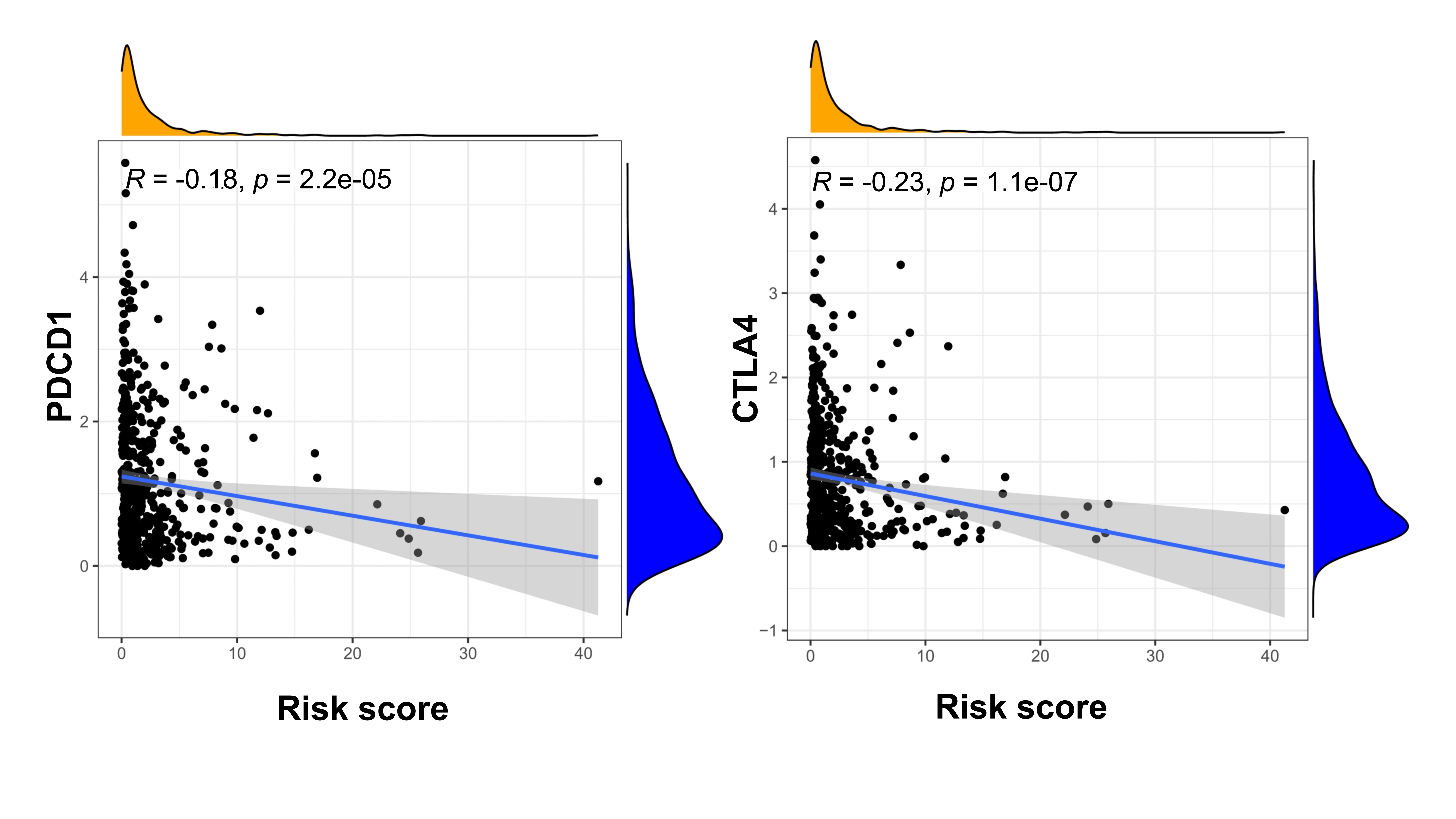

Supplement: Supplementary file 2 [file Image1.JPEG]

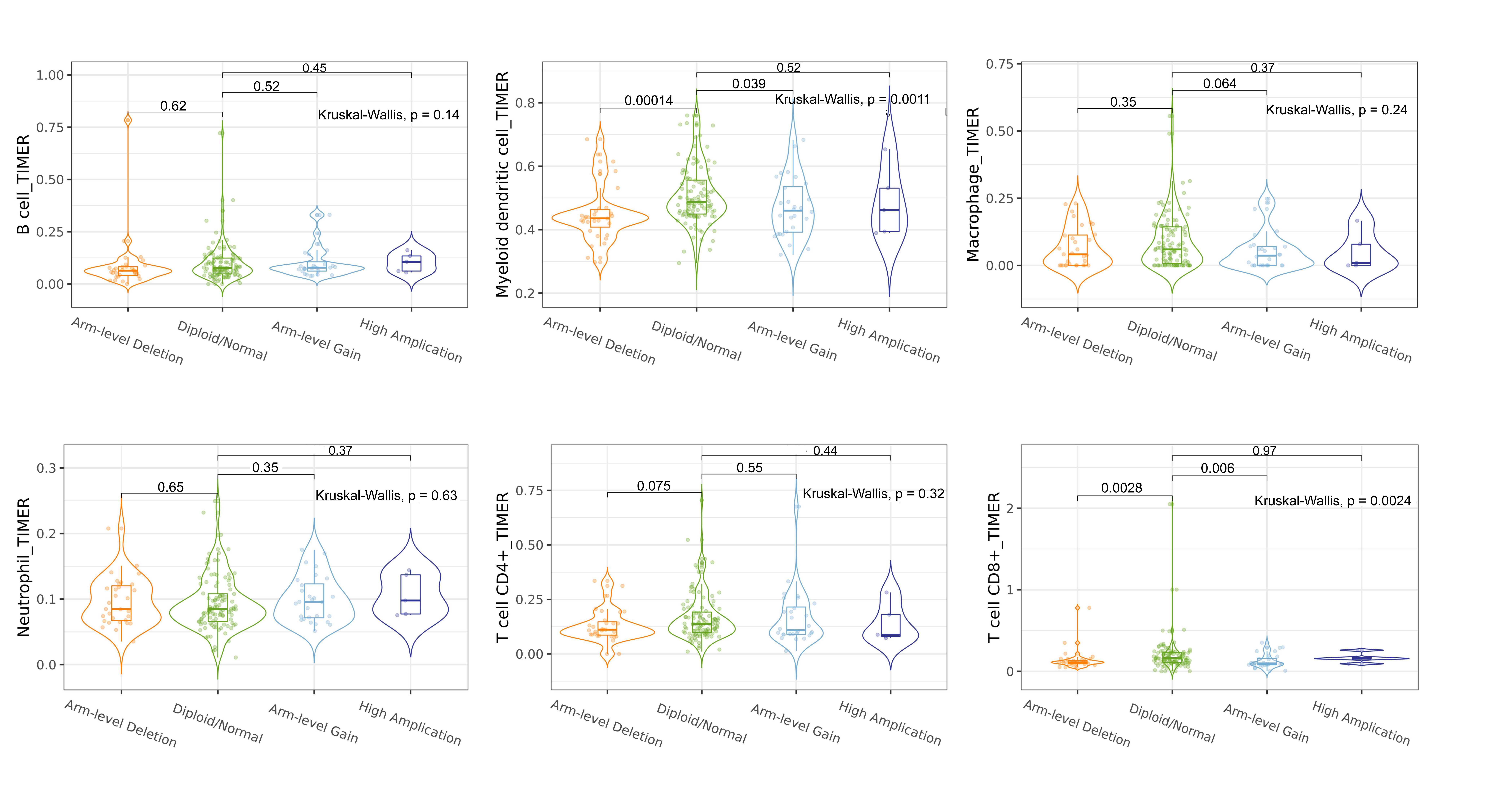

Supplement: Supplementary file 3 [file Image4.JPEG]

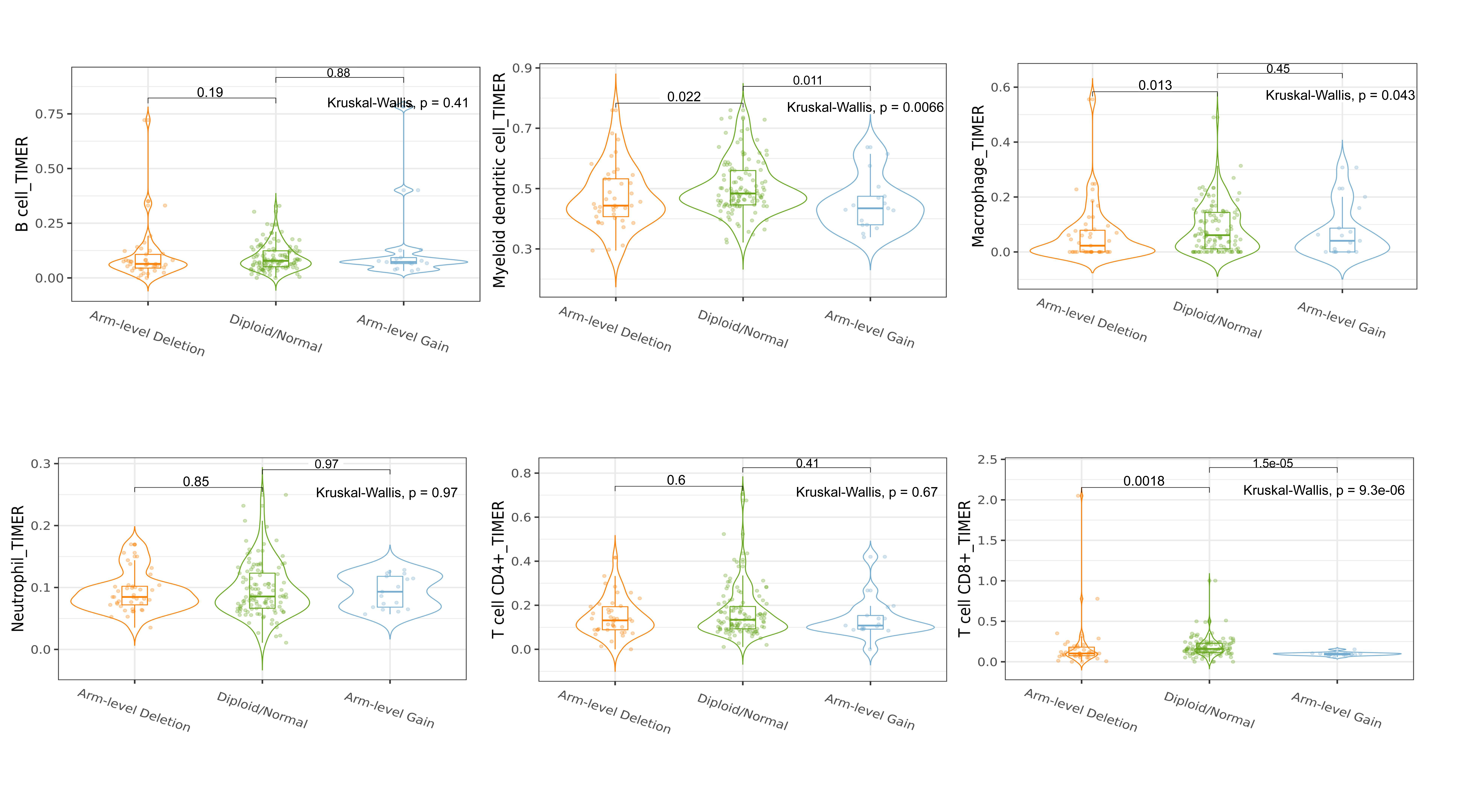

Supplement: Supplementary file 5 [file Image5.JPEG]
